# The Relationship Between Postgraduates’ Emotional Intelligence and Well-Being: The Chain Mediating Effect of Social Support and Psychological Resilience

**DOI:** 10.3389/fpsyg.2022.865025

**Published:** 2022-06-14

**Authors:** Zhang Shuo, Deng Xuyang, Zhao Xin, Cai Xuebin, Hou Jie

**Affiliations:** ^1^Center of Mental Health Education, Yangzhou University, Yangzhou, China; ^2^Center of Mental Health Education, Southeast University, Nanjing, China; ^3^College of Educational Sciences, Yangzhou University, Yangzhou, China; ^4^College of Electrical, Energy and Power Engineering, Yangzhou University, Yangzhou, China

**Keywords:** postgraduates, emotional intelligence, social support, psychological resilience, well-being

## Abstract

**Background:**

Postgraduates usually face more life challenges than undergraduate students, including social, emotional and financial issues, and the prevalence of mental health problems in postgraduates is higher than undergraduates. Therefore, the attention on postgraduates’ mental health status is needed.

**Objectives:**

The current study explored the relationship between postgraduates’ emotional intelligence and well-being by investigating the mediating effects of social support and psychological resilience and the relationship between them through the construction of a chain mediation model.

**Method:**

1,228 postgraduates completed the Emotional Intelligence Scale, the Social Support Rate Scale, the Psychological Resilience Scale, and the Subject Well-being Scale.

**Results:**

There is a chain effect between postgraduates’ social support and psychological resilience mediated by their emotional intelligence and well-being, with a total effect value of 0.049.

**Conclusion:**

Emotional intelligence has a predictive effect on postgraduates’ well-being. The mechanism of this effect includes the indirect effects of social support and psychological resilience. Study results revealed the relationship mechanism between emotional intelligence and postgraduates’ well-being, and provide reference for explorations of how to development postgraduates’ emotional intelligence and further improving their abilities to strengthen their emotional resilience.

## Introduction

Currently, there is an increasing number of postgraduate enrollments in Chinese higher education institutions. In 2021, the National Bureau of Statistics reported that there are 3.33 million graduate students currently enrolled, with 1.18 million new enrollments (National Bureau of Statistics of China, 2022). The increasing postgraduate enrollments in China may challenge the graduation education and management of higher education institutions. Comparing to undergraduate students, postgraduates students are more competent in education and professional skills, however, they may face profound pressures on economic, academic, family, interpersonal relationship and employment. Due to the life challenges that postgraduates may encounter, postgraduates are more susceptible to mental health issues. Study found that postgraduates do have a higher incidence of interpersonal and mental health problems (Luo et al., 2015). Also, comparing to undergraduate students, postgraduates have higher suicide rates (Feng et al., 2015). Nowadays, postgraduates’ mental health and well-being has begun to attract increasing attention from researchers, for example, research has shown that the detection rate of mental health problems of postgraduates is 12.3% (Ye et al., 2017), among which the three most common psychological problems postgraduates face are compulsion, depression, and interpersonal sensitivity (Zhou, 2018). Anxiety is also a common disorder among postgraduates (Feng and Zhang, 2017), with the pressures experienced reflected primarily through sleep, emotional, and social interaction issues (Song et al., 2019). There are relatively limited researches focus on the group of postgraduates worldwide, which could be explained by that outside of China, postgraduates may cover a wider age span and more variety in status. The outbreak of COVID-19 is another crucial crisis event for college students, which severely affected the well-being of college students, the prevalence of depressive symptoms during the pandemic in college students is 26.0%, and significantly higher prevalence in postgraduates than in undergraduates (Luo et al., 2021).

Well-being refers to one’s evaluation of satisfaction and happiness of quality of their current lives, in that a higher sense of well-being entails an individual experiencing more positive emotion and fewer negative emotions (Diener et al., 1999). It is a happy state of mind (Huang et al., 2012), which encompasses one’s overall evaluation of their existing sense of quality of life and mental health, according to their own standards of well-being (Zhu and Liu, 2019). Well-being plays an important role in motivating individuals to improve their individual quality of life and mental health, maintain interpersonal harmony, and shape a rational and peaceful social mentality. For postgraduate students, well-being reflects their evaluation of quality of life, and has an impact on their ability to adapt in life, and further their academic research and career development. Research has shown that the well-being level of postgraduates is significantly lower than that of undergraduates (Jiang and Chen, 2020).

Emotional intelligence refers to an individual’s ability to process emotional information accurately and effectively. It is a skill developed through both learning and experience which, at its core, is about how to perceive, use, understand, and manage emotions (Mayer et al., 1999). People with high emotional intelligence can effectively identify emotions in daily interpersonal communication – both their own and others’ – producing positive attitudes and then make appropriate emotional and behavioral responses. In doing this, these individuals then experience a higher level of mental health as well as garnering more social support (Huang and Dai, 1997; Zeidner et al., 2009). Bar-On’s emotional social intelligence model proposes that emotional intelligence is a combination of the effective understanding of one’s self and others, getting along well with others, and successful adaptation and responses to one’s environment, eventually bringing about a sense of well-being (Xu and Zhang, 2002; Bar-On, 2010). Studies have shown that emotional intelligence plays a key role in well-being (Gallagher and Vella-Brodrick, 2008; Mavroveli et al., 2011; Wang and Cai, 2015; Liang and Wang, 2018; Li, 2020). Individuals with high emotional intelligence have been shown to be better at perceiving, regulating, and controlling their actions and responses, experience a more positive emotional experience in their surrounding environment, feel a higher level of life satisfaction, and are better able to create and achieve stronger levels of well-being (Higgs and Dulewicz, 2014). This is especially evident in younger age groups such as college students (Wu et al., 2016).

Ecological System Theory proposes that an individual exists in a series of mutual influences, nested in an environmental system. EST has been long discussed by scholars to link biological, psychological and social environments. According to the Bronfenbrenner’s theory, there are five systems of ecological model, each contained within the next level of system. The five systems are interrelated, so the human Development is then the result of interactions between individual factors and ecological environment factors. The first level is Microsystem, relationship in this level is bi-directional, which means individual could be influenced by others also have capacity to influence others. The microsystem refers to the parents, peers, schools and etc. Second level is mesosystem, which refers the connections or interactions between the microsystems, such as the connections between the family, teachers and peers. Third level is exosystem, involving the social structures do not contain individuals themselves, for example, parents’ workplaces, parent’s friends and mass media. The fourth level is macrosystem, focuses on the cultural elements, such as socioeconomic status and ethnicity. The final level is chronosystem, consists of all environment events and transitions over the lifetime. Based on the concepts of ECT, it is necessary to explore the factors that affect the well-being of postgraduates not only at the individual level, but also with consideration of interaction mechanisms at play with the ecological environment (Zhou et al., 2020). The current study explores the influential patterns of emotional intelligence on postgraduates’ well-being, includes microsystem level structures.

Self-determination theory, which proposed by the Edward Deci and Richard Ryan, refers to each person’s ability to make decisions and manage their own life (Deci and Ryan, 2008). This theory demonstrated that motivation is the most basic intrinsic drive for individuals to complete any tasks or behave. Higher self-determination represents more internal form of motivation, and low self-determination associates with more external form of motivation and amotivation. SDT suggests that there are three psychological needs: autonomy, the need of sense of feeling control of their own behaviors and goals; competence, the need of gaining master of tasks and skills; relatedness, the need of sense of belonging and attach to other people. Research has shown that individuals who are autonomously will exhibit better emotional regulation and integration of negative affect (Weinstein and Hodgins, 2009). Emotional Competence is also an important part for well-being, Kotsou et al. (2011) investigated the training program to enhance the emotional competence, and find that emotional competence significantly related to physical and subjective well-being. Further, the social support also aligns with another domain of SDT, the need for relatedness. Previous study already demonstrated the association of social support and mental health (Poudel et al., 2020). Another theory similar to the SDT is Bandura’s self-efficacy theory, which defines as people’s capacities to control over their own functioning, which contains four domains: mastery experiences, vicarious experiences, social persuasion and emotional states. The differences of SDT and SET is that SDT suggests that the feeling of competence, autonomy and relatedness is a more distal factor to behavior, which focuses more on the motivation, on the contrary, self-efficacy has a more direct influence on behaviors (Sweet et al., 2012). Both theories will further suggest the emotional intelligence and social support are key elements of well-beings.

H1. Postgraduates’ emotional intelligence can positively predict well-being.

Existing studies have focused more on the relationship between emotional intelligence and well-being, with other researches limited to exploring the intermediary role of variables such as self-esteem and interpersonal trust, and lacked mechanism analysis of the internal relationship of other factors (such as social support, psychological resilience, etc.). Social support, as an important component of social relations (Hakulinen et al., 2016), refers to the emotional, financial, material, and other forms of resource support provided by others (e.g., family, friends, classmates, teachers, neighbors) when one is in need. Social support plays an important protective role in maintaining individual mental health, alleviating and improving bad emotions (Li et al., 2017). A strong level of social support reflects a harmonious relationship between an individual and the micro-systems surrounding them, promoting positive personality formation, cognitive development and social adaptation (Zhang et al., 2019). In the case of postgraduates, one’s social support system has been found to be the first way many are able to lower psychological pressures (Song et al., 2019). The Main Effect Model of social support notes that regardless of whether an individual is in a state of stress or not, one’s sense of social support has a positive effect on their life, enhancing the individual’s sense of well-being (Cohen, 2004). Social support can both improve positive emotions and reduce negative emotions (Uchino et al., 1996), and has a positive impact on one’s perceived level of well-being (Luo and Mu, 2017). There is also a close relationship between social support and emotional intelligence. People with high emotional intelligence tend to feel more subjective and objective social support and make better use of it (Zhu, 2006). This means that such individuals have stronger skills in social adaptability, which improves the quality of their interpersonal relations and therefore their level of well-being. This has been confirmed by numerous studies which have explored social support as an intermediary variable (Zeidner and Matthews, 2016; Xiao and Hou, 2017; Dehghani, 2018). Ecosystem Theory suggests that social support, as an external positive protective factor for the development of postgraduates’ level of well-being, plays an intermediary role in the relationship between emotional intelligence and subjective well-being (He et al., 2020).

H2. Postgraduates’ social support plays an intermediary role in the relationship between emotional intelligence and well-being.

Psychological Resilience is a psychological process in which individuals make use of many positive factors to maintain and develop their physical and mental health to find well-being in their lives when they are faced with various pressures, setbacks, or difficulties (Wang and Wang, 2013). Some studies have found that a considerable number of postgraduates lack resilience and self-confidence, with the proportion of postgraduates with low psychological resilience accounting for 15% (Gong et al., 2020). Meanwhile, the relationship between psychological resilience and negative emotions appears to be regulated by age, with the association stronger in adults than in children or adolescents (Hu et al., 2015), further highlighting the need to focus on postgraduates’ psychological resilience. Cultivating psychological resilience in young academics can lessen academic stress, simultaneously enhancing their levels of physical and mental health and increasing their positive emotions (Liu et al., 2015; An and Pei, 2017). Research has also found a significant positive correlation between life satisfaction, positive emotion, and psychological resilience, and psychological resilience is shown to have a strong positive predictive effect on one’s well-being (Yang et al., 2012). People with high emotional intelligence have a higher tolerance for life changes, setbacks, and adversity, and show fewer negative behaviors which indicates that have stronger levels of psychological resilience. Emotional intelligence is therefore an important factor affecting postgraduates’ psychological resilience (Wen et al., 2014). If postgraduates can effectively perceive, understand, and adjust their internal emotions, and, when affected by strong emotional fluctuations, are nonetheless able to quickly and smoothly return to a normal psychological state, they are better able to muster more positive energy internally to deal with the difficulties they face.

H3. Postgraduates’ psychological resilience plays an intermediary role in the relationship between emotional intelligence and well-being.

Psychological Resilience Theory asserts that one’s personal abilities and traits are not the only determinants of psychological resilience, but that external factors such as family support and social support also play an important role (Olsson et al., 2003). Psychological resilience involves an interaction with environments of positive social support which contribute to improvements in psychological resilience (Bradley et al., 2013). Studies have confirmed that social support can be a protective factor for psychological resilience (Glozah and Pevalin, 2014), with positive family relationships enabling adolescents to obtain even more positive feedback from their families (Wu et al., 2014), and positive peer and teacher-student relationships also enhancing students’ psychological resilience (Morrison and Allen, 2007). When faced with adversity, they can then demonstrate more resilience because of their sufficient inner senses of security and self-confidence. This indicates a strong interaction between social support and psychological resilience, and we infer that there is an active mechanism between postgraduates’ social support and psychological resilience in the relationship between emotional intelligence and well-being. Specifically, we expected that postgraduates with high emotional intelligence are able to make better use of social supports to deal with psychological imbalances and stressors such as issues in interpersonal communication, academic research pressures, career anxiety, and maintaining stable and harmonious communication with others, which leads to them being able to attain a more fulfilling level of well-being.

H4. Postgraduates’ social support and psychological resilience plays a chain mediating role in the relationship between emotional intelligence and well-being.

Existing research into the emotional lives of postgraduates has largely focused on levels of emotional intelligence, while the interactions between the factors of emotional intelligence, well-being, social support, and psychological resilience are also of profound significance. The current study will further analyze the internal mechanism of emotional intelligence on well-being in an attempt to provide a scientific basis for education on postgraduates’ well-being, and to improve mental health educators’ understandings of the importance of addressing and developing postgraduates’ emotional intelligence, social support, and psychological resilience in practice, in order to enhance postgraduates’ senses of well-being.

## Materials and Methods

### Participants

Convenience sampling was used to test 1,228 postgraduates from across three universities in Jiangsu Province. A online questionnaire was used to collect data, with 1,140 valid questionnaires received. The sample mean age was 23.73 (*SD* = 1.88).

### Variables and Measures

#### Emotional Intelligence

The Emotional Intelligence Scale (ELS) was used, as developed by Schutte et al., and adapted by Wang (2002). The ELS is considered to be one of the most representative scales in emotional intelligence research. The scale comprises 33 items addressing four dimensions: perception of emotion, regulation of self-emotion, regulation of others’ emotion, and use of emotion. Using a five-point Likert scoring method, the higher the total score, the higher one’s level of emotional intelligence. In the current study, the Cronbach’s α was 0.935.

#### Well-Being

Well-being was measured using the Chinese version of the Index of Well-Being (IWB) as compiled by Campbell to measure subjects’ degree of well-being experienced (Li and Zhao, 2000). The measure is done into two parts: the general affective index scale and the life satisfaction questionnaire. The former consisted of eight items that examine one’s perceptions of emotion in different ways; the latter consists of only one item. In the current study, final scores ranged from 2.1 (least happy) to 14.7 (most happy), and the Cronbach’s α was 0.948.

#### Social Support

Xiao’s (1994) Social Support Rating Scale (SSRS) was used to measure subjects’ perceived degree of social support. This scale comprises 10 items and three dimensions: subjective support, objective support, and utilization of support. The higher the score, the higher one’s perceived social support. In order to achieve relevance to the characteristics of postgraduates in early adult life, some items were revised in the current study, such as using the terms “classmates” instead of “colleagues”, or “lovers” instead of “spouse.” In this study, the Cronbach’s α was 0.806.

#### Psychological Resilience

Psychological Resilience was measured using the Chinese version of the Connor-Davidson Resilience Scale (CD-RISC) as translated by Yu and Zhang (2007). This measure comprises 25 items and three dimensions: tenacity, strength, and optimism. Each is ranked using a five-point Likert scale. The higher the total score, the higher one’s level of psychological resilience. The Cronbach’s α in the current study was 0.971.

### Data Analysis

SPSS 21.0 and Amos 26.0 were used to analyze the data collected in the current study.

## Results

### Common Method Deviation Analysis

Harman’s single factor test was used to exclude common method deviation caused by the questionnaire method. The results showed that there were 19 factors with eigenvalues greater than 1, and the variation explained by the first factor was 26.58% which is less than the critical value of 40%, indicating that the effect of common method deviation would not affect our data results.

### Relationship Between Emotional Intelligence, Social Support, Psychological Resilience, and Well-Being

A significant positive correlation was found between emotional intelligence, social support, psychological resilience, and well-being (Table 1).

**TABLE 1:** Means, standard deviations, and correlations for study variables (*N* = 1,140).

		*M*	*D*	1	2	3	4
1	Emotional intelligence	129.17	16.14	1			
2	Social support	40.39	7.54	0.295***	1		
3	Psychological resilience	93.88	15.68	0.646***	0.388***	1	
4	Well-being	11.31	2.58	0.307***	0.461***	0.458***	1

***p < 0.001.

### Emotional Intelligence and Well-Being: Chained Mediating Analyses

**FIGURE 1:**
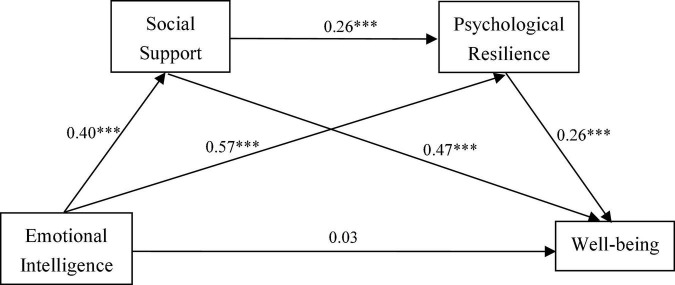
Chain-mediation model of emotional intelligence, social support, psychological resilience and well-being. ***p < 0.001.

According to the model 6 in the Process program developed by Hayes (2013), a chain mediation model was established with emotional intelligence as an independent variable, social support and psychological resilience as mediating variables, and well-being as a dependent variable (Figure 1). The model showed a satisfactory fit to the data, χ^2^/*df* = 4.81, RMSEA = 0.06, CFI = 0.98, TLI = 0.97. As shown in the diagram, emotional intelligence significantly and positively predicted social support (β = 0.40, *P* < .001), social support significantly positively predicted psychological resilience (β = 0.26, *P* < .001), and psychological resilience significantly and positively predicted well-being (β = 0.26 *P* < .001).

The bootstrapping method was used to re-sample 5,000 times to calculate for a 95% CI. As shown in Table 2, the results showed that social support and psychological resilience played an intermediary role between emotional intelligence and well-being, and the total mediating effect was 0.049, while the 95% CI was [0.040, 0.060]. The indirect effect on the emotional intelligence→social support→well-being path was 0.016, while the 95% CI was [0.012, 0.021]. The emotional intelligence→social support→psychological resilience→well-being path was 0.003, while the 95% CI was [0.002, 0.005]. The indirect effect on the emotional intelligence→psychological resilience→well-being path was 0.030, while the 95% CI was [0.023, 0.039]. Additionally, the results showed that the 95% CIs of the indirect effects differed significantly from zero, and that the mediating effects had statistical significance.

**TABLE 2:** Chain mediating effect on emotional intelligence, social support, psychological resilience, and well-being (*N* = 1,140).

Model pathways	Model explanatory quantity		Estimated effect	95% CI	
	*R* ^2^	*F*		Lower bounds	Upper bounds
*Mediating effect*					
Emotional intelligence → social support → well-being	0.245	183.942***	0.016	0.012	0.021
Emotional intelligence → social support → psychological resilience → well-being	0.304	165.409***	0.003	0.002	0.005
Emotional intelligence → psychological resilience → well-being	0.210	150.834***	0.03	0.023	0.039
*Total mediating effect*			0.049	0.04	0.06

***p < 0.001.

## Discussion

The current study focused on Chinese postgraduates, constructing a chain mediation model based on ecosystem theory, and examined the chain mediating role of social support and psychological resilience between emotional intelligence and well-being. The findings showed that the research hypothesis was supported.

### Emotional Intelligence and Well-Being

The results of the current study showed that postgraduates’ emotional intelligence significantly and positively predicted their well-being, which is consistent with the results of previous studies (Wang et al., 2018). According to Bar-On’s theory, emotional intelligence significantly impacts well-being (Bar-On, 2005). As mature young adults, postgraduates with high emotional intelligence are better able to realize their own and other people’s emotions, and will also be better at expressing and managing their own emotions, allowing them to have a sharper perception of their surrounding environment. Higher emotional intelligence can also improve communication abilities between postgraduates and their teachers in both academic research and sharing emotional experiences, enabling them to receive more positive feedback, maintain good energy levels in both their study and personal lives, thereby reporting more positive emotional experiences and maintaining a stronger sense of well-being in their everyday life. Conversely, postgraduates with lower emotional intelligence are more likely to suppress their negative emotions causing them to not be able to correctly perceive their true feelings toward others. They therefore have weaker abilities to deal with social relations, experiencing more negative emotions as a result which can lead to self-doubt and a lower sense of overall well-being.

### The Mediating Effect of Social Support

The current study found that postgraduates’ social support had a mediating effect between emotional intelligence and well-being, showing that emotional intelligence contributed to a higher level of perceived social support. Social support was a positive experience which promoted the improvement of postgraduates’ level of well-being. As an individual in early adulthood, postgraduates have a strong need to develop social relations (Arnett, 2000) and those with high emotional intelligence can better perceive, understand, and experience the emotions involved in social relations. High emotional intelligence also allows them to better perceive and accept social support offered by their family, friends, and other contacts, allowing them to effectively use the support of others so as to maintain their own levels of mental health and well-being, promoting the bridging role of social support between emotional intelligence and well-being. The positive experience of well-being is highly rewarding, further promoting individuals to repeatedly seek out behaviors or activities that bring further well-being (Snyder and Shane, 2006). This finding has also been verified by Zeidner and Matthews (2016), whose research suggested that social support plays an important role in emotional intelligence and well-being (included in the umbrella of mental health), providing clear evidence for focusing on and enhancing the organic cycle of emotional intelligence and well-being. The result is also consistent with the previous study conducted by Lopez-Zafra et al. (2019), which proves that social support mediates the relationship of emotional intelligence and psychological well-being in Moroccan adolescents. Previous researchers also indicated that social support mediates the relationship between emotional intelligence and other psychological outcomes, for example, social support can partial mediate the relationship between emotional intelligence and occupational stress (Valenti et al., 2021) and mediate the relationship between emotional intelligence and worry (Zysberg and Zisberg, 2022). In the current study, social support was shown to play a significant intermediary role, indicating that postgraduates’ level of well-being was not only related to personal characteristics such as emotional intelligence, but also closely related to external environmental factors such as social support density, which was consistent with ecosystem theory.

### The Mediating Effect of Psychological Resilience

Although emotional intelligence and social support are important influencing factors on postgraduates’ well-being, findings showed that individuals with similar levels of social support nonetheless had different abilities in adapting to adversity, trauma, or other major life pressures. Therefore, it was necessary to explore the mediating role of psychological resilience between emotional intelligence and well-being. Compared with undergraduates, postgraduates face heavier academic research pressure, burdened with more practical tasks in both their academic and personal lives, as well as pressures regarding job hunting or career development. Individual cognitive levels have been shown to develop gradually in terms of being able to cope with these difficulties, with a “qualitative” leap that is often a part of human development. Qiu (2002) found that the age of this qualitative leap tends to take place when one is 22 or 23 years old, which is often the beginning of one’s period as a postgraduate. Psychological resilience therefore plays an important role during this period of time. Previous studies have shown that psychological resilience can effectively increase one’s positive emotions, enhancing both their physical and mental health, and improving their levels of well-being and life satisfaction (Waugh et al., 2011; Liu et al., 2012; Liu et al., 2015). The previous research has indicated that resilience is a predictor of healthy psychological state which can play a partial mediating role between emotional intelligence and psychological well-being (Akbari and Khormaiee, 2015). The current study further promotes this idea and found that a higher level of emotional intelligence brought about a more stable sense of one’s psychological resilience in life, helping postgraduates to better manage their emotions and deal with frustrations and making it easier to deal with problems and difficulties in a positive way. The higher postgraduates’ level of psychological resilience, the more optimistic they were about many things in life, making them more capable and resilient when facing difficulties. Similarly, the more positive emotions they experienced, the higher their level of life satisfaction, which then feeds back into the loop by continuously supporting and promoting them in satisfying their psychological needs.

### The Chain-Mediating Effect of Social Support and Psychological Resilience

The results of the current study show that postgraduates’ emotional intelligence had an impact on their well-being through the chain mediation of social support and psychological resilience. Our results support the proposals put forth by Ecosystem Theory, in that the impact of the environment on individuals varies as a result of particular characteristics (Ding et al., 2017). Postgraduates with higher emotional intelligence and with an external environment of high social support were more likely to engage in positive and effective behaviors and strategies to deal with heavy pressures, thereby improving their levels of self-efficacy and self-confidence (Bandura et al., 1999). This allows for the continuous development of their psychological resilience and adaptability to difficulties, enabling them to internalize a more stable and positive psychological state. With a foundation of higher levels of life satisfaction and self-identity, these postgraduates are then able to receive more positive feedback, further promoting their interpersonal skills in social communication. This cycle of enhancement and reinforcement of positive behavior constantly strengthens their internal psychological state, leading to more stable emotional intelligence and psychological resilience, allowing them to constantly adjust and develop new internal mechanisms for well-being. This chain-mediation of social support and psychological resilience is realized in a positive and meaningful way, leading these postgraduates to experience a greater level of well-being.

### Practical Implications and Limitations

This study introduced two variables – social support and psychological resilience – which expands on the existing research into the impact of emotional intelligence on individual well-being. Emotional intelligence plays a role in maintaining positive interpersonal relationships and can effectively help postgraduates obtain necessary social connections and social support (Zijlmans et al., 2013). Meanwhile, negative emotions such as loneliness or helplessness can also be reduced, improving psychological health and well-being through the accumulation of more high-quality social capital (Aka and Gencoz, 2014). Research into the status and influencing factors of postgraduates’ well-being is helpful in that it reveals ways in which it can be supported or improved, as well as highlighting the mechanisms to consider when developing interventions. First, a more holistic approach is needed that considers the roles of emotional intelligence and other personal traits when attempting to improve postgraduates’ levels of well-being in order to create positive space for them to develop = better daily management patterns and skills. Furthermore, when mentoring postgraduates in how to negotiate emotional changes or difficulties, students should be encouraged and guided in how to better express their emotional experiences. Second, a beneficial social environment should be created that matches the psychological needs of postgraduates that will improve their academic social support networks, with consideration of making tutors, teachers, and administrative personnel available, as well as education them in how to effectively perceive and fully utilize the social support they have. This will enhance postgraduates’ positive emotional experiences and further improve their mental health levels. Finally, universities should not only provide a positive external environment for postgraduates, but also make efficient use of the different roles of individual characteristics and behavior differences within the social environment which can affect individual well-being. Especially in considering the development mechanism of postgraduates’ psychological resilience, activities that encompass rich content and a variety of forms should be made available to postgraduates, and their involvement should be encouraged. It is important to pay attention not only to postgraduates’ research and academic skills, but also to have regular dialog on a broad range of topics (such as personal interests, psychology, etc.) to promote more holistic psychological growth and, therefore, more positive emotions, encouraging a stronger sense of autonomy in their self-development and self-realization.

Several limitations of this study should be considered. First, this study used a cross-sectional research design to explore the mechanism of emotional intelligence on well-being. Cross-sectional research can explain or explore causal relationships between variables, in part, but future studies should use a longitudinal tracking design to further test and confirm the results of the current study. Second, our data collection was done through postgraduates’ self-reports. Future research should make use of multiple data collection means, more channels, and using more comprehensive methods to conduct more comprehensive research. Third, social support and psychological resilience were shown to play an important role in postgraduates’ daily lives and learning experiences. However, it’s possible that other internal personal traits or external factors can also affect the well-being level of postgraduates. Future research should explore and test other potential variables which could impact postgraduates’ experiences. For example, psychological resilience is also linked with social class, resilience also emerges in negative family and social context, researchers could further take account the influence of social context variables in the study of postgraduates.

## Conclusion

The current study reveals a significant positive correlation between the emotional intelligence and well-being of postgraduates, and that social support plays an intermediary role between emotional intelligence and well-being, while psychological resilience mediates between emotional intelligence and well-being. Social support and psychological resilience mediate the chain between emotional intelligence and well-being.

## Data Availability Statement

The original contributions presented in this study are included in the article/supplementary material, further inquiries can be directed to the corresponding author.

## Ethics Statement

The studies involving human participants were reviewed and approved by the Ethics Committee for the School of Educational Sciences, Yangzhou University. The patients/participants provided their written informed consent to participate in this study.

## Author Contributions

ZS contributed to conception, design of the study and wrote the first draft of the manuscript. ZX and HJ collected the raw data and organized the database. CX performed the statistical analysis. DX revised it critically for important intellectual content. All authors contributed to the article and approved the submitted version.

## Conflict of Interest

The authors declare that the research was conducted in the absence of any commercial or financial relationships that could be construed as a potential conflict of interest.

## Publisher’s Note

All claims expressed in this article are solely those of the authors and do not necessarily represent those of their affiliated organizations, or those of the publisher, the editors and the reviewers. Any product that may be evaluated in this article, or claim that may be made by its manufacturer, is not guaranteed or endorsed by the publisher.
